# The role of hypoglycemia in the burden of living with diabetes among adults with diabetes and family members: results from the DAWN2 study in The Netherlands

**DOI:** 10.1186/s12889-018-5064-y

**Published:** 2018-01-18

**Authors:** Giesje Nefs, François Pouwer

**Affiliations:** 1 CoRPS - Center of Research on Psychological and Somatic disorders, Department of Medical and Clinical Psychology, Tilburg University, PO BOX 90153, 5000 LE Tilburg, the Netherlands; 2 Diabeter, National treatment and research center for children, adolescents and young adults with type 1 diabetes, Rotterdam, The Netherlands; 3 Radboud university medical center, Radboud Institute for Health Sciences, Department of Medical Psychology, Nijmegen, The Netherlands; 4 Department of Psychology, University of Southern Denmark, Odense, Denmark

**Keywords:** Hypoglycemia, Worries, Burden, Family members

## Abstract

**Background:**

To examine the relation between self-reported hypoglycemic events, worries about these episodes, and the burden of diabetes in adults with diabetes and family members from The Netherlands.

**Methods:**

As part of the second multinational Diabetes Attitudes, Wishes and Needs (DAWN2) study, 412 Dutch adults with type 1 or type 2 diabetes and 86 family members completed questions about the burden of living with diabetes, the frequency of hypoglycemia, worries about these events, and several demographic and clinical factors. Analyses included hierarchical logistic regression.

**Results:**

In total, 41% of people with diabetes and 56% of family members considered diabetes at least somewhat of a burden. In people with diabetes, diabetes burden was independently associated with self-reported current insulin use (fully adjusted OR = 2.75, 95% CI 1.49–5.10), self-reported frequent non-severe hypoglycemia in the past year (OR = 2.45, 1.25–4.83), self-reported severe hypoglycemia in the past year (OR = 1.91, 1.02–3.58), and being very worried about hypoglycemia at least occasionally (OR = 3.64, 2.18–6.10). For family members, the odds of experiencing living with diabetes as a burden was increased only for participants who were at least occasionally very worried about hypoglycemia (adjusted OR = 5.07, 1.12–23.00).

**Conclusions:**

Approximately half of adults with diabetes and adult family members experienced at least some diabetes burden. In both groups, diabetes burden appeared to be associated with being very worried about hypoglycemia at least occasionally. If these results are replicated, new intervention studies could test new ways of decreasing the traumatic consequences of previous or anticipated hypoglycemic events for people with diabetes and family members.

## Background

Despite ongoing improvements in technology and care, living with diabetes remains a challenge for many people. The second Diabetes Attitudes, Wishes and Needs (DAWN2) study among 8596 adults with diabetes across 17 countries found that 12% of participants reported poor or very poor overall quality of life, 14% had likely depression and 45% showed high diabetes-specific distress [1]. In addition, diabetes had a negative impact on all examined aspects of life, including physical health and relationships [1]. Diabetes and its treatment not only affect the person living with this condition, but may also have an impact on family members [2]. The DAWN2 study surveyed 2057 adult family members and found that over one third perceived a considerable burden of diabetes on the family [3]. People with diabetes and their family members may experience a range of worries about risks connected with diabetes, with hypoglycemia featuring as one of the key themes (Hermanns et al., submitted; [4]).

Hypoglycemia remains the greatest barrier to achieving and maintaining tight glycemic control in people with type 1 diabetes and people with type 2 diabetes on insulin therapy or sulfonylurea treatment [5]. The prevalence of severe hypoglycemia (requiring assistance from another person for recovery [6]) varies according to diabetes type and treatment duration, ranging from 7% in people with type 2 diabetes using sulfonylurea or insulin for less than 2 years to 46% in those with long-standing type 1 diabetes (> 15 years) [7]. Non-severe hypoglycemia (events manageable by the person with diabetes) is also an important problem as symptoms can be unpleasant and aversive, and may interfere with following treatment recommendations [8, 9].

Findings of the international DAWN2 study among adults with diabetes and family members of people with diabetes suggest psychological outcomes are worse when hypoglycemia is more frequent [10, 11]. However, substantial between-country variation in outcomes and associations in these studies underlines the need for country-specific analyses [10, 11]. Therefore, the present study used data from the Dutch sample of the DAWN2 study to examine the relation between the frequency of hypoglycemia, worries about these events, and the burden of diabetes in adults with diabetes and family members from The Netherlands.

## Methods

### Procedure and participants

The second Diabetes Attitudes Wishes and Needs (DAWN2) study used a cross-sectional questionnaire survey in 17 countries across four continents, to examine the attitudes, wishes and needs of people with diabetes, family members and healthcare professionals [12]. Design and procedures for the Dutch sub study were in line with the international study protocol [12]. For the sample of people with diabetes, inclusion criteria were age ≥ 18 years, a diagnosis of (non-gestational) diabetes by a healthcare professional at least 12 months ago, and disclosure of current diabetes treatment. For the sample of family members, inclusion criteria were age ≥ 18 years, not having diabetes yourself, living in the same household with an adult with (non-gestational) diabetes diagnosed at least 12 months ago, and being involved in their diabetes care. Potential participants were initially identified via existing panels and databases. Individuals were then contacted via email or by phone. Interviews were conducted online (self-administered), by phone, or face-to-face (for a small number). The study was conducted in accordance with ethical requirements and all participants provided informed consent.

The Dutch subsamples included 502 adults with diabetes and 120 family members. For the present analyses, we excluded people taking injectables other than insulin (“non-insulin injectables”, e.g. GLP-1 agonists) as there were relatively few of them (*n* = 15) and their data might cloud the results. We also excluded people not meeting the definition of type 1 and type 2 diabetes as employed in DAWN2 [1]. Type 1 diabetes was defined as self-reported (a) diagnosis before the age of 30, (b) prescription of insulin when first diagnosed, and (c) current insulin use. Type 2 diabetes was defined as self-reported (a) diagnosis on or after the age of 30, and (b) no prescription of insulin when first diagnosed. This left 412 people with diabetes and 86 family members.

### Measurements

#### Diabetes burden

As a proxy of diabetes burden in people with diabetes, we used an item from the Problem Areas in Diabetes questionnaire [13]. Participants were asked to indicate on a five-point Likert scale ranging from “not a problem” to “serious problem” whether “Feeling that diabetes is taking up too much of your mental and physical energy every day” was currently a problem for them. Diabetes was considered a burden if participants reported a minor, moderate, somewhat serious, or serious problem. We preferred the pooling of people with minor and major concerns over the pooling of people with minor and no concerns, as this last approach would ignore a group who do report a problem (albeit not of major proportions). Family members were asked to indicate on a five-point Likert scale ranging from “no burden” to “very large burden” how much of a burden it was for them to help manage the diabetes of the person they lived with. Diabetes was considered a burden if participants reported a slight, moderate, large or very large burden.

#### Hypoglycemic events

People with diabetes and family members were asked to report the average number of times the person with diabetes has had symptoms of hypoglycemia, which they could treat themselves during the past 12 months. Based on clinical experience and the distribution of replies, frequency of non-severe hypoglycemia was categorized as “none” (no symptoms in past 12 months), “occasional” (once a month, less often than once a month) and “frequent” (at least once a day, at least once a week, several times a month). In addition, both groups were asked to report approximately how many times during the past 12 months the person with diabetes has had severely low blood sugar that he/she was unable to treat himself/herself and needed help from someone to restore blood sugar levels. Severe hypoglycemia during the past 12 months was categorized as “no” versus “one or more events”.

#### Worries about hypoglycemia

People with diabetes and family members were asked to rate on a four-point Likert scale to what extent they agreed with the statement “I am very worried about the risk of [him/her having] hypoglycemic (low blood sugar) events”. When they mainly disagreed, mainly agreed and fully agreed with the statement, this was taken to indicate the presence of at least some worries about hypoglycemia.

#### Demographic and clinical covariates

For both people with diabetes and family members, variables included gender, age, educational level (with a high level defined as having completed higher vocational education or university), working full or part-time (no/yes), and whether the person with diabetes was using insulin to manage diabetes (no/yes). For people with diabetes, additional information included whether they were currently living with somebody (no/yes), diabetes duration, and the presence of one or more (potentially diabetes-related) co-morbid conditions (stroke, foot ulcer, foot/leg amputation, kidney disease, eye damage, nerve damage, heart disease).

### Statistical analyses

Two separate hierarchical (blockwise) logistic regression analyses were used (one for people with diabetes and one for family members) to examine the association of hypoglycemic events and worries about hypoglycemia with diabetes burden. For family members, step 1 included gender, age, educational level, employment, insulin use, non-severe hypoglycemia and severe hypoglycemia. In step 2, worries about hypoglycemia were added. For people with diabetes, living situation, diabetes duration and co-morbidities were also included in step 1. All analyses were performed using SPSS Version 22 (IBM SPSS Statistics, New York). A *p*-value < 0.05 indicated statistical significance.

## Results

### Sample characteristics

Descriptive characteristics of the samples of adults with diabetes and family members are displayed in Table 1. Compared to people with diabetes, family members appeared to be more likely to be female, to have a high educational level, and to be employed, and were somewhat younger. In the sample of people with diabetes, participants with type 1 diabetes were somewhat overrepresented compared to national estimates [14]. People with diabetes were somewhat more likely to report frequent non-severe hypoglycemia, while family members more commonly indicated that the person with diabetes they lived with had experienced severe hypoglycemia during the past 12 months. Approximately 60% of people with diabetes and family members were at least occasionally very worried about hypoglycemia, while living with diabetes was a small to large burden for half of participants in both groups.

**Table 1:** Descriptive characteristics of the sample of adults with diabetes and adult family members

	Missing values	Adults with diabetes (*n* = 412)	Missing values	Family members (*n* = 86)
Female gender	0	48% (197)	0	83% (71)
Age, years	0	60 ± 11 (62, 54–67)	0	54 ± 13 (56, 50–62)
High educational level	17	26% (102)	3	35% (29)
Living alone	0	27% (113)		NA
Working full- or parttime	2	27% (112)	0	38% (33)
Diabetes type and treatment	0		0	
Type 2 diabetes, no medication		22% (91)		15% (13)
Type 2 diabetes, oral medication only		36% (150)		51% (44)
Type 2 diabetes, insulin		22% (91)		26% (22)
Type 1 diabetes		19% (80)		8% (7)
Diabetes duration, years	0	13 ± 12 (9, 4–17)		NA
Co-morbidities	0			NA
No		45% (184)		
One		30% (125)		
Two or more		25% (103)		
Non-severe hypoglycemia in past year	3		5	
None		33% (134)		40% (32)
Occasional		32% (132)		36% (29)
Frequent		35% (143)		25% (20)
Severe hypoglycemia in past year (≥1 event)	1	20% (83)	22	30% (19)
Worries about hypoglycemia	19	57% (225)	7	61% (48)
Diabetes burden	0	41% (170)	0	56% (48)

*NA* not applicable/available

Values are % (n) or mean ± SD (median, interquartile range)

### Diabetes burden in people with diabetes

When the frequency of non-severe and severe hypoglycemia were entered in step 1 along with the demographic and clinical covariates, this first model was statistically significant (*χ*^*2*^(12) = 72, *p* < 0.001; Cox and Snell R square = 0.17; Nagelkerke R square = 0.23). As shown in Table 2, participants who used insulin (*p* = 0.003), who experienced frequent non-severe hypoglycemia during the past 12 months (compared to people reporting none of these events; *p* < 0.001) and who had experienced a severe hypoglycemic event during the past 12 months (*p* = 0.02) had higher odds of considering diabetes at least somewhat of a burden, adjusted for the other variables in model 1. Entry of worries about hypoglycemia in step 2 led to a significant model improvement (change *χ*^*2*^(1) = 26, *p* < 0.001; Cox and Snell R square = 0.23; Nagelkerke R square = 0.31). In this fully adjusted model (*χ*^*2*^(13) = 98, *p* < 0.001), participants who used insulin (*p* = 0.001), who experienced frequent non-severe hypoglycemia during the past 12 months (compared with people reporting none of these events; *p* = 0.009), who had experienced a severe hypoglycemic event during the past 12 months (*p* = 0.04), and who were at least occasionally very worried about hypoglycemia (*p* < 0.001) had higher odds of considering diabetes at least somewhat of a burden.

**Table 2:** The relation between demographic, clinical and psychological factors and the burden of diabetes

	Adults with diabetes (*n* = 377)		Family members (*n* = 58)	
	Model 1	Model 2	Model 1	Model 2
Female gender	1.04 (0.64–1.69)	1.08 (0.65–1.79)	1.24 (0.23–6.87)	1.00 (0.16–6.12)
Age, years	0.98 (0.95–1.01)	0.98 (0.95–1.01)	0.99 (0.94–1.04)	0.98 (0.93–1.03)
High educational level	0.92 (0.53–1.58)	1.09 (0.62–1.94)	0.53 (0.13–2.11)	0.99 (0.21–4.66)
Living alone	1.24 (0.74–2.09)	1.23 (0.71–2.10)	NA	NA
Working full- or parttime	0.70 (0.39–1.24)	0.66 (0.36–1.20)	1.67 (0.43–6.44)	0.98 (0.22–4.35)
Diabetes duration, years	0.99 (0.97–1.01)	0.99 (0.96–1.01)	NA	NA
Using insulin	**2.45 (1.36–4.41)**	**2.75 (1.49–5.10)**	1.57 (0.37–6.77)	0.86 (0.17–4.35)
Co-morbidities			NA	NA
No	Ref	Ref		
One	1.36 (0.79–2.35)	1.36 (0.77–2.38)		
Two or more	1.00 (0.54–1.86)	1.04 (0.55–1.98)		
Non-severe hypoglycemia in past year				
None	Ref	Ref	Ref	Ref
Occasional	1.75 (0.95–3.23)	1.33 (0.70–2.52)	1.15 (0.26–5.06)	0.69 (0.13–3.53)
Frequent	**3.55 (1.87–6.76)**	**2.45 (1.25–4.83)**	3.84 (0.65–22.79)	1.52 (0.20–11.34)
Severe hypoglycemia in past year (≥1 event)	**2.00 (1.09–3.66)**	**1.91 (1.02–3.58)**	1.51 (0.34–6.78)	2.28 (0.44–11.87)
Worries about hypoglycemia		**3.64 (2.18–6.10)**		**5.07 (1.12–23.00)**

*NA* not applicable/available

Hierarchical logistic regression analyses. Values are OR (95% CI); bold = statistically significant (*p* < 0.05)

### Diabetes burden in family members

When the frequency of non-severe and severe hypoglycemia were entered in step 1 along with the demographic and clinical covariates, this first model was not statistically significant (*χ*^*2*^(8) = 8, *p* = 0.42; Cox and Snell R square 0.13; Nagelkerke R square 0.18). As shown in Table 2, none of the demographic and clinical variables in this step were independently associated with diabetes burden in family members. Entry of worries about hypoglycemia in step 2 led to a significant model improvement (change *χ*^*2*^(1) = 5, *p* = 0.03; Cox and Snell R square = 0.20; Nagelkerke R square = 0.27). However, this fully adjusted model was still not statistically significant (*χ*^*2*^(9) = 13, *p* = 0.16). Family members who were at least occasionally very worried about hypoglycemia (*p* = 0.04) had higher odds of considering diabetes at least somewhat of a burden, adjusted for the other variables in model 2.

## Discussion

This study based on the Dutch subsample of the international DAWN2 study found that almost half of adults with diabetes considered diabetes at least somewhat of a burden. A direct comparison with figures from other countries is difficult to make. While we based the definition of diabetes burden on one item focusing on the mental and physical costs of living with this condition, the cross-national benchmarking paper of the DAWN2 study considered several more global measures including the composite score of the Problem Areas in Diabetes 5 item version and the DAWN Impact of Diabetes Profile [1]. Countries ranked differently across these different indicators of diabetes burden, where no individual country or region appeared to be consistently better or worse than the others [1]. Overall, the Dutch sample appeared to be among the quarter of countries with the most optimal scores in this domain [1]. With respect to family members, we found that more than half of the Dutch participants indicated at least some burden in helping the person with diabetes they lived with managing their diabetes. The international DAWN2 papers employed a more strict definition of burden, finding 35% of the total sample reporting a moderate to very large burden [3], with the Dutch subsample scoring slightly under the total mean [3].

In participants with diabetes, we found a positive association between self-reported insulin use and experienced diabetes burden, adjusted for the other variables. Previous studies have suggested that insulin therapy is viewed as the most burdensome treatment modality among people with type 2 diabetes [15]. While negative appraisals of insulin therapy are highest among those who do not have previous experience with insulin therapy, people who are currently using insulin or have prior experience with this treatment modality still experience substantial negative views about insulin [15–17]. In the present study, the relation between self-reported insulin use and diabetes burden was independent of worries about hypoglycemia and self-reported occurrence of severe and non-severe hypoglycemic events in the previous year, suggesting other aspects of insulin treatment might play a role. For example, in the Diabetes MILES – Australia study half of participants with type 2 diabetes using insulin believed that taking insulin meant their diabetes had become worse and that insulin caused weight gain [16]. Furthermore, approximately 25% of adults with type 1 or insulin-treated type 2 diabetes experience injection-related anxiety [18, 19]. In addition, both of these groups frequently have to cope with actual or anticipated negative attention and judgment from others when self-injecting insulin by pen or pump [20–22].

With respect to hypoglycemia, previous studies have mostly focused on the psychological impact of severe events, finding a relation with greater diabetes-related distress and poorer general emotional well-being [23]. A large European study among people with type 1 diabetes and insulin-treated type 2 diabetes showed that non-severe hypoglycemia may also have a substantial negative impact on energy and mood [24]. In the present study, people with diabetes also had increased odds of diabetes burden not only when they had experienced at least one self-reported severe hypoglycemic event, but also in case of self-reported frequent non-severe hypoglycemia during the past 12 months, adjusted for the other variables. The relation between proxy-reported hypoglycemic events and diabetes burden was not found in family members. This may be explained by the relatively small number of family members available for the regression analysis. Alternatively, it is possible that family members have not been aware of all hypoglycemic events that have occurred. From previous studies, it is known that people with diabetes and their family members often disagree about the frequency and nature of hypoglycemic episodes [25, 26].

In both people with diabetes and family members, more worries about hypoglycemia were associated with higher diabetes burden, adjusted for the other variables. While the recent occurrence of hypoglycemic events has been associated with higher worries about hypoglycemia [23, 27, 28], the relation between worries about hypoglycemia and diabetes burden was independent of the occurrence of self- or proxy-reported severe and non-severe hypoglycemic events in the past year. Several studies from the pediatric diabetes literature suggest that, apart from the frequency of events, the types of experiences with hypoglycemia (e.g. with seizures or unconsciousness) may determine the level of experienced worries about future events [29–31]. A study among 90 adults with type 1 diabetes found that over 25% met diagnostic criteria of hypoglycemia-specific posttraumatic stress and reported that perceived threat of death from hypoglycemia and fear of hypoglycemia were significantly related to these symptoms, while number of recent hypoglycemic episodes was not [32]. From the scientific literature on posttraumatic stress we know that it is also possible to experience symptoms triggered by anticipatory thoughts about future distressing events [33]. In family members, the extent to which they believe their relative with diabetes is able to cope with hypoglycemic episodes may also be an important determinant of the level of experienced worries about hypoglycemia. This hypothesis is supported by literature from the pediatric diabetes field, where trait anxiety levels and recent experiences with hypoglycemia predicted fear of hypoglycemia in adolescents with type 1 diabetes but beliefs about whether their child carried emergency glucose were associated with fear of hypoglycemia in parents [29].

The findings of this study should be considered in terms of its limitations and strengths. First, while cross-country variations in outcomes necessitate the additional analysis for specific countries, the number of participating family members from the Netherlands was relatively low. This may have significantly underpowered the present analyses. Similarly, the number of participants in the present study does not allow stratifying the analyses for diabetes type. Also, unfortunately, the distribution of replies on the items about burden and worries and the increased number of analyses did not allow sensitivity analyses in which different definitions were used. Second, we had to rely on self- or proxy report about clinical variables such as diabetes type. Our decision to adhere to the previously used DAWN2 definitions of type 1 and type 2 diabetes based on age of diagnosis, insulin use shortly after diagnosis, and current insulin use [1] may have safeguarded against some of the biases inherent to self- or proxy report. However, we may have wrongfully excluded participants who would have qualified as type 1 or type 2 diabetes based on laboratory data, but diverge from the standard clinical picture on which the DAWN2 diabetes definitions were based. Furthermore, in this questionnaire-based study no objective clinical data (e.g. glycemic control, use of continuous glucose monitor) was available. Third, we used different definitions of diabetes burden in people with diabetes and family members, which makes a direct comparison of prevalence and correlates across these groups more difficult. Fourth, the main measures were based on single purpose-designed items rather than validated questionnaires. The one-item question from the PAID has not been officially validated as a proxy for diabetes burden, but was selected based on experience with the measure in clinical practice. Fifth, as one reviewer eloquently stated, “correlation does not mean causation”. While we make some suggestions to guide intervention in the following paragraph, an experimental design is needed to establish whether manipulating worries about hypoglycemia actually lowers perceived disease burden. Finally, the results (especially for family members) should be interpreted with caution in light of several statistical considerations. Given the exploratory nature of the study, we did not adjust for multiple testing, with increased risk of type 1 errors. In addition, the number of observations is small relative to the number of predictors, increasing the risk of overfitting. The regression analysis for family members was run with a relatively high number of predictors to ensure optimal comparability with the analysis in people with diabetes, but it is prudent to repeat the analysis based on the larger international family members data set before strong conclusions are drawn. Also, even though we found a significant association between worries about hypoglycemia and diabetes burden in family members, it should be kept in mind that the test of the overall null for family members was not statistically significant. Strengths of the study include the focus on both people with diabetes and adult family members and the diverse demographic, (albeit self-reported) clinical and psychological correlates that were considered.

Most existing educational programs and behavioral interventions in the field of hypoglycemia to date have primarily focused on the avoidance of future events or improved awareness. These have generally found a reduction in the number of hypoglycemic episodes and an improvement in awareness, while improvements in worries about hypoglycemia were less consistent [34–37]. New technologies such as continuous glucose monitoring and sensor-augmented pump therapy have shown some promise in reducing hypoglycemia-related worries, but results are inconsistent and a more thorough evaluation of their potential with respect to patient-reported outcomes is warranted [38]. As memories of previous traumatic hypoglycemic events may be difficult to overcome, a focus on the prevention and management of hypoglycemia may not be enough to reduce fear of hypoglycemia for all adults with diabetes [38]. In this context, the anxiety and stress reducing properties of eye movement desensitization and reprocessing treatment should be explored further [33]. Interventions could also target worries about hypoglycemia in adult family members, as these may lead to suboptimal psychological outcomes of family members as well as suboptimal diabetes support [4, 11].

## Conclusions

In conclusion, approximately half of adults with diabetes and adult family members experienced at least some diabetes burden. In both groups, being very worried about hypoglycemia at least occasionally appeared to be independently associated with diabetes burden. Other significant independent correlates with higher diabetes burden in people with diabetes included self-reported insulin use, self-reported frequent non-severe hypoglycemia and self-reported severe hypoglycemia. The merits of new technologies and educational / behavioral interventions with a primary focus on the prevention of hypoglycemic events and improved hypoglycemia awareness in reducing fear of hypoglycemia need to be explored further. However, these could be supplemented with intervention studies that test new treatments targeting the traumatic consequences of previous or future anticipated events and hypoglycemia (self)-efficacy. The role and worries of family members may also be considered. Given the disappointing reproducibility of scientific findings [39], replication of results is an important first step in this process.

## Abbreviations

DAWN2 study: the second Diabetes Attitudes, Wishes and Needs study
